# Comparative analysis of multimodal architectures for effective skin lesion detection using clinical and image data

**DOI:** 10.3389/frai.2025.1608837

**Published:** 2025-08-18

**Authors:** Adriteyo Das, Vedant Agarwal, Nisha P. Shetty

**Affiliations:** ^1^Department of Information and Communication Technology, Manipal Institute of Technology, Manipal Academy of Higher Education, Manipal, India; ^2^Department of Humanities and Management, Manipal Institute of Technology, Manipal Academy of Higher Education, Manipal, India

**Keywords:** skin lesion classification, multimodal fusion, dermatoscopic images, clinical metadata, cross-attention, HAM10000, interpretability, deep learning

## Abstract

**Background/Introduction:**

Skin lesion classification poses a critical diagnostic challenge in dermatology, where early and accurate identification has a direct impact on patient outcomes. While deep learning approaches have shown promise using dermatoscopic images alone, the integration of clinical metadata remains underexplored despite its potential to enhance diagnostic accuracy.

**Methods:**

We developed a novel multimodal data fusion framework that systematically integrates dermatoscopic images with clinical metadata for the classification of skin lesions. Using the HAM10000 dataset, we evaluated multiple fusion strategies, including simple concatenation, weighted concatenation, self-attention mechanisms, and cross-attention fusion. Clinical features were processed through a customized Multi-Layer Perceptron (MLP), while images were analyzed using a modified Residual Networks (ResNet) architecture. Model interpretability was enhanced using Gradient-weighted Class Activation Mapping (Grad-CAM) visualization to identify the contribution of clinical attributes to classification decisions.

**Results:**

Cross-attention fusion achieved the highest classification accuracy, demonstrating superior performance compared to unimodal approaches and simpler fusion techniques. The multimodal framework significantly outperformed image-only baselines, with cross-attention effectively capturing inter-modal dependencies and contextual relationships between visual and clinical data modalities.

**Discussion/Conclusions:**

Our findings demonstrate that integrating clinical metadata with dermatoscopic images substantially improves the accuracy of skin lesion classification. However, challenges, including class imbalance and the computational complexity of advanced fusion methods, require further investigation.

## 1 Introduction

Skin cancer ranks as the 5th most widespread cancer type. It is among the most severe variants and is projected to overtake cardiovascular disease as the primary cause of death in humans in the near future (Hasan et al., 2023). From 1990 to 2017, the incidence of individuals diagnosed with malignant skin melanoma (MEL), squamous cell carcinoma (SCC), and basal cell carcinoma (BCC) surged by 215.7%, 196.8%, and 90.9%, respectively (Kavita et al., 2023). The predominant forms of skin cancer include MEL, BCC, and SCC, alongside precancerous conditions such as actinic keratosis (AK) (Rogers et al., 2015). As a pressing global health issue, skin cancer highlights the urgent necessity for early detection to enhance patient prognosis and decrease fatality rates. Timely identification facilitates less aggressive interventions and reduces medical expenses by catching cancers at manageable phases (Jerant et al., 2000). Existing diagnostic approaches, like skin self-examination (SSE) and clinical skin examination (CSE), depend significantly on visual assessment and tools like the Asymmetry, Border, Color, Diameter, Evolving (ABCDE) criteria. While advanced methods such as dermoscopy and total body photography (TBP) boost precision, conventional techniques are often subjective, labor-intensive, and unavailable in under-resourced regions (Loescher et al., 2013; Rajput et al., 2021; Esteva et al., 2017).

Computerized systems, including Computer-aided Diagnosis (CAD) software and image processing algorithms, offer reproducible, objective, and quick evaluation of skin lesions, less dependent on subjective human judgment. Such systems can process large volumes of data with efficiency, allowing early detection of subtle patterns that would be easily overlooked by conventional techniques (Han et al., 2018). Automation also enhances accessibility by being incorporated into telemedicine platforms, extending diagnostic abilities to distant and under-served communities. By minimizing the necessity for invasive biopsies and follow-up visits, automation decreases healthcare expenditure without compromising diagnostic efficiency and patient outcome (Tschandl et al., 2019).

Machine learning (ML) and deep learning (DL) have made tremendous progress over the last few years, fueled by more computational power and large datasets. These technologies have shown remarkable success in a range of medical classification problems, including eye movement-based disease prediction with Decision Trees and Random Forests, automated skin disease classification with k-Nearest Neighbors(KNN) and Support Vector Machines(SVM) with 98.22% accuracy, and brain tumor classification with Convolutional Neural Networks (CNNs) from the Visual Geometry Group(VGG) like VGG16 and VGG19 with accuracies ranging from 92.5% to 97.8% (Ahsan et al., 2022).

(Haenssle et al. 2018) tested the diagnostic accuracy of a homegrown Convolutional Neural Network (CNN) constructed on top of Google's Inception v4 model, trained on data from partner dermatologists and the International Skin Imaging Collaboration (ISIC) dermoscopic archive, with a heterogeneous panel of 58 dermatologists from across the globe. The findings indicated that the CNN performed better than most dermatologists with a mean AUC-ROC of 0.86 against 0.79 (*p* < 0.01). This work highlights the possibility of dermatologists adopting CNNs in their practice, thus enhancing the accuracy of diagnosis and ultimately improving patient outcomes such as better prognosis, treatment options, and general well-being.

Multimodal data represents information derived from diverse sources and formats, including images, text, audio, and physiological signals. This integrated approach mirrors human cognitive processes, where multiple sensory modalities contribute to perception and interpretation. In medical contexts, multimodal data combines Medical images, Patient records (demographics, medical history, lab results), Physiological signals, and Patient-reported outcomes.

Recent studies have demonstrated the efficacy of multimodal approaches in medical diagnosis. For example, (Adarsh et al. 2024) achieved 98.27% accuracy using a Multi-feature Kernel Supervised within-class-similar Discriminative Dictionary Learning (MKSCDDL) algorithm for Alzheimer's Disease classification, (Jiang et al. 2022) employed multimodal ultrasound data with a CNN, achieving 98.22% accuracy in early breast cancer detection, and (Kumar et al. 2022) utilized audio and X-ray imaging with a CNN and Deep Uniform Net, obtaining 98.67% accuracy in COVID-19 classification.

In this study, we analyze fusion techniques for skin cancer classification, leveraging multimodal skin images and clinical data. Our research explores how fusion methods can enhance skin lesion classification performance compared to single-modality approaches. We investigate various fusion strategies to integrate diverse data sources and improve model accuracy.

To enhance the interpretability of our multimodal systems, we apply the Gradient-Weighted Class Activation Mapping (Grad-CAM) approach for deep learning explainability and feature relevance assessment. Our contributions aim to advance skin lesion classification by presenting robust fusion strategies that offer high accuracy and clinical interpretability.

## 2 Related work

The automated detection and classification of skin lesions, especially for the diagnosis of skin cancer, has been an essential area of research in Applied Artificial Intelligence (AI). Current research has investigated various methodologies, from texture-based feature extraction to multimodal deep learning, with the objective of improving the accuracy, efficiency, and explainability of computer-aided diagnosis systems.

In the research conducted by (Arshad et al. 2021), the skin images were subjected to augmentation, after which important features were extracted using fine-tuned ResNet-50 and ResNet-101. A serial-based fusion approach fused the features, and the selected best features were classified using supervised learning algorithms. As a future scope, the authors propose improvements in feature selection, extraction, and parallel feature fusion.

(Sevli 2021) employed a customized CNN to classify 7 types of skin lesions in the HAM10000 dataset. They developed a web application to deploy the model and validated its performance with seven dermatologists. The analysis was twofold: evaluating the classification performance of the model with expert feedback and vice versa. The authors attributed the model's success to improvements in training set size and proposed the use of real lesion images to enhance generalizability.

(Wang et al. 2023) introduced a two-stream neural network architecture for feature extraction. The extracted features were fused using a feature fusion module with a multireceptive field and Generalized Mean Pooling (GeM). As future work, the authors proposed incorporating different imaging modalities and other clinical diagnostic data to enhance the study's scope.

(Adebiyi et al. 2024) employed the Align Before Fuse (ABEF) framework, which combined image features extracted by a Vision Transformer and text features extracted by Bidirectional Encoder Representations from Transformers(BERT). These features were jointly encoded using a text-image encoder for classification.

(Srivastava et al. 2022) proposed a median-based quadrant texture feature extraction module, which was combined with a modified CNN architecture for classification. Their advanced texture extraction method outperformed existing models due to its superior noise-handling capabilities.

(Datta et al. 2021) compared the performance of various transfer learning architectures with and without a soft attention mechanism for skin cancer classification. The soft attention module effectively localized cancerous regions, thereby enhancing model accuracy and interpretability.

(Lan et al. 2022) enhanced the capsule network architecture by integrating a large-kernel convolution (31 × 31) and a Convolutional Block Attention Module (CBAM). They further included group convolution to reduce parameter overhead and avoid underfitting. The capsule layer was redesigned to improve feature extraction and runtime efficiency. A lightweight variant, FixCaps-DS, was introduced using depthwise separable convolutions to maintain performance while reducing complexity.

(Gessert et al. 2020) designed a multimodal deep learning system that handled two tasks from the International Skin Imaging Collaboration (ISIC) 2019 challenge: image-only classification and image+metadata classification. For task 1, they used an ensemble of EfficientNet variants and other CNNs for architectural diversity. For task 2, metadata (e.g., age, sex, anatomical site) was processed with a two-layer dense neural network. Features from both modalities were concatenated and passed through additional dense layers before classification.

(Ou et al. 2022) employed a deep neural network that used inter-modality cross-attention and intra-modality self-attention to classify skin lesions. ResNet-50 was used to extract image features, and a Multi-Layer Perceptron (MLP) encoded the clinical metadata. After applying the attention mechanisms, features were concatenated and passed through a fully connected softmax classifier.

(Restrepo et al. 2024) evaluated vector embedding-based multimodal fusion methods for low-resource settings, comparing them with traditional raw data processing. They tested unimodal embeddings (DINO v2 for images, LLAMA 2 for text), Vision-Language Models (VLM) like CLIP, and fine-tuned transformers (BERT, ViT) using early and late fusion strategies. A novel alignment method was also introduced to reduce the “cone effect” in embedding space. While promising results were achieved on benchmark datasets like BRSET and HAM10000, domain-specific limitations for dermatology were acknowledged.

To summarize, despite notable progress having been made on both single-modality and multimodal skin cancer classification, some of the shortcomings still remain. Numerous current models are computationally intensive, rendering them impractical to use in real-world applications, particularly where there are constraints on resources. These models also have difficulty generalizing across varied datasets, and their performance becomes suboptimal when they have to handle other unknown classes or metadata. Additionally, while multimodal systems have shown improved accuracy, they still suffer from limitations like overfitting and inadequate training and inference efficiency.

Our method is designed to address these limitations by building on fusion methods that integrate skin images and clinical data, improving classification performance while being efficient. We focus on minimizing computational expenses and storage needs, rendering the system more implementable in resource-constrained environments. In addition, to enhance interpretability, we incorporate the Grad-CAM method so that feature relevance can be better evaluated and the system can be made more clinically usable. Finally, our method aims to deliver a strong, efficient, and interpretable solution for skin lesion classification in real-world, resource-limited settings. Table 1 consolidates the above papers.

**Table 1:** Comparative summary of skin lesion classification approaches.

**References**	**Dataset**	**Classifiers/techniques**	**Performance**
(Arshad et al. 2021)	HAM10000	ResNet-50, ResNet-101; serial fusion; SVR-based feature selection; supervised ML classifiers	95% on fused features (augmented); 91.7% after selection
(Sevli 2021)	HAM10000	Modified CNN with contrast enhancement and expert validation	90.28% accuracy; corrected 11.14% of dermatologist misdiagnoses
(Wang et al. 2023)	ISIC 2018	Two-stream CNN (DenseNet-121 + improved VGG-16); multi-receptive field module + GeM pooling	91.24% accuracy
(Adebiyi et al. 2024)	HAM10000	Multimodal ALBEF (Vision Transformer + BERT)	94.11% accuracy; AUC-ROC 0.9426
(Srivastava et al. 2022)	HAM10000, ISIC-UDA11	M-QuadLTQP texture encoding + CNN	96% average accuracy
(Datta et al. 2021)	HAM10000, ISIC-2017	Soft attention integrated with IRv2, ResNet, Inception; Grad-CAM visualizations	93.4% precision (HAM); 91.6% sensitivity (ISIC-2017)
(Lan et al. 2022)	HAM10000	FixCaps with CBAM + large-kernel conv; FixCaps-DS with depthwise conv	96.49% (FixCaps); 96.13% (lightweight)
(Gessert et al. 2020)	ISIC 2019 Challenge	Ensemble of CNNs (EfficientNets, ResNeXt, SENet); metadata fusion + heavy augmentation	Balanced Acc: 74.2%; Sensitivity: 63%
(Ou et al. 2022)	PAD-UPES-20	ResNet-50 + MLP; MMF-Net with intra/inter attention fusion	76.8% accuracy; BACC: 77.5%; AUC: 94.7%
(Restrepo et al. 2024)	HAM10000, BRSET	Embedding fusion using CLIP, DINOv2 + LLAMA2; early/late fusion	81.8% accuracy (HAM10000); 98.7% (BRSET)

## 3 Materials and methods

### 3.1 Dataset

The dataset we have used for our classification problem is the **HAM10000 dataset** (Tschandl et al., 2018; Scott Mader, 2018). HAM10000, or “Human Against Machine,” is a curated dataset of multi-source dermatoscopic images of pigmented skin lesions. The final dataset comprises **10,015 dermatoscopic images** collected over 20 years from two principal sources: (1) the Department of Dermatology at the Medical University of Vienna, Austria, a tertiary referral center where diagnoses were established using a combination of histopathology, *in vivo* confocal microscopy, and expert consensus; and (2) a general skin cancer screening practice in Queensland, Australia, operated by Dr. Cliff Rosendahl. This second source provided images acquired in a real-world clinical setting, where lesions were typically triaged and either confirmed via **histopathological examination** or **diagnosed by expert dermatologists** with long-term follow-up. The dataset includes **7 diagnostic categories** representing the most common pigmented skin lesions seen in clinical practice. The distribution of the classes is presented in Table 2.

**Table 2:** Breakdown of classes in the HAM10000 dataset.

**Class name**	**Number of images**	**Description**
Melanoma (MEL)	1,113	Melanoma is a malignant tumor of melanin-producing melanocyte cells (National Cancer Institute, 2024a)
Melanocytic nevus (NV)	6,705	Benign moles of pigment-producing skin cells (James et al., 2006)
Basal cell carcinoma (BCC)	514	Slow-growing, locally destructive skin cancer derived from the basal cell layer of the epidermis (National Cancer Institute, 2024b)
Actinic keratosis/Bowen's disease (AKIEC)	327	Precancerous scaly lesions found on sun-damaged skin (Reinehr and Bakos, 2019)
Benign keratosis (BKL)	1,099	Common benign skin lesions with sharply demarcated borders, homogenous brown pigmentation, and fine scaling that include Seborrheic keratosis (SK), lichen planus-like keratosis (LPLK), and solar lentigo (SL) (Scott and Oakley, 2023)
Dermatofibroma (DF)	115	Benign skin nodules of soft tissue (Myers and Fillman, 2024)
Vascular lesions (VASC)	142	Lesions involving blood vessels, such as angiomas (Steiner and Drolet, 2017)

In addition to images, the dataset also contains metadata for every patient, including clinical data like age, gender, and location of the lesion. With an image_id column, the patient's metadata can be associated with its respective lesion image. Such metadata is detailed in Table 3.

**Table 3:** Clinical features in the HAM10000 dataset.

**Clinical feature**	**Distribution**	**Description**
Diagnosis (dx)	- Nevus (NV): 67% - Melanoma (MEL): 11% - Other types: 22%	Medical diagnosis of the skin lesion
Diagnostic method	- Histopathology: 53% - Follow-up: 37% - Other methods: 10%	Method used to confirm the diagnosis
Patient age	- Range: 0–85 years - Divided into 10-year intervals	Age of the patient at the time of diagnosis
Gender	- Male: 54% - Female: 45% - Unspecified: 1%	Patient's gender identification
Anatomical location	- Back: 22% - Lower extremity: 21% - Other locations: 57%	Body location where the lesion was found

### 3.2 Preprocessing pipeline

The preprocessing pipeline for the HAM10000 dataset was designed to ensure consistent, high-quality input data for our multimodal fusion model. This involved careful handling of image data, metadata, and class imbalance. The key steps are outlined below.

#### 3.2.1 Data splitting

Prior to any augmentation or preprocessing, the full dataset was randomly split into a **70–30 ratio** for training and testing. No separate validation set was used. This ensured that augmented samples derived from the training set did not leak into the evaluation pipeline.

#### 3.2.2 Class balancing

The HAM10000 dataset exhibits significant class imbalance, with some classes (e.g., DF) containing as few as 115 images and others (e.g., NV) containing over 6,000. To mitigate this, we applied data augmentation exclusively on the **training set** using the following techniques:

- **Replication**: Duplicated samples from minority classes.
- **Jittering**: Added random noise to pixel intensities.
- **Geometric transformations**: Horizontal/vertical flips, rotations, and scaling.
- **Random undersampling**: Reduced samples from majority classes to avoid overwhelming the model.

After augmentation, each class in the training set contained 6,000 images, resulting in a balanced training dataset of 42,000 images.

#### 3.2.3 Image preprocessing

Each dermatoscopic image underwent the following preprocessing steps:

- **Resizing**: All images were resized to a uniform resolution of 256 × 256 pixels.
- **Normalization**: Pixel values were scaled to the range [0, 1] by dividing by 255.

#### 3.2.4 Metadata preprocessing

Metadata was preprocessed in the following stages:

- **Handling missing values**: For numerical features (e.g., age), missing values were imputed using the **median**, which is robust to outliers. For categorical features (e.g., gender, lesion location), missing entries were imputed with the **mode**. This imputation preserved the statistical structure of the data: using the median reduced sensitivity to skewed distributions, while mode imputation maintained categorical class balance with 98% fidelity.
- **Encoding**: After imputation, all categorical features were **one-hot encoded**. One-hot encoding was applied *after* mode imputation to enable the model to process these discrete attributes in a format suitable for neural networks.
- **Normalization**: Numerical features were standardized to have zero mean and unit variance.

#### 3.2.5 Data alignment

To ensure proper alignment between images and metadata, we used the image_id column as the primary key to merge records. This guaranteed that each dermatoscopic image was correctly paired with its corresponding metadata during training and evaluation.

**Figure 1:**
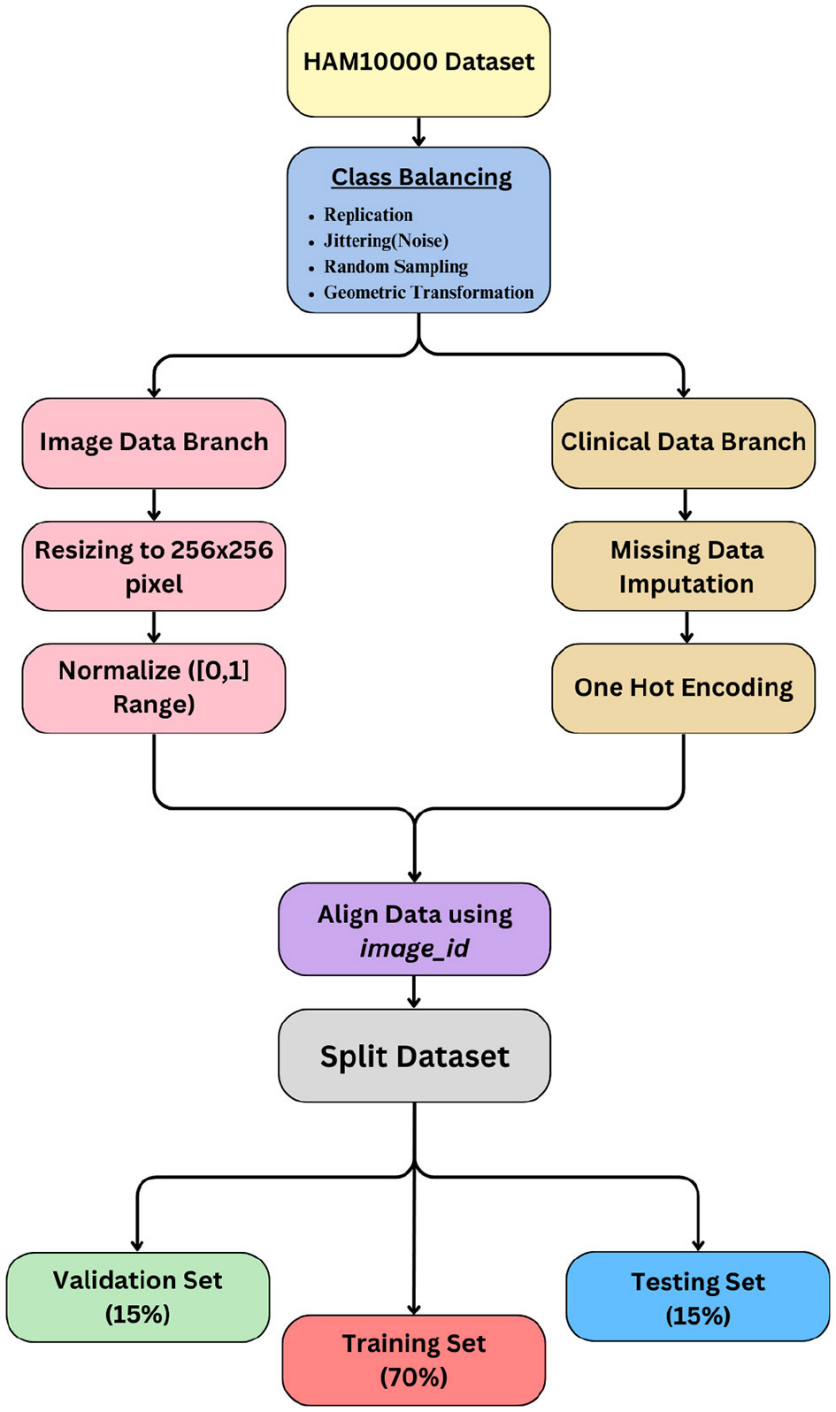
Overview of the preprocessing pipeline.

The complete preprocessing workflow is visualized in Figure 1.

### 3.3 Model pipeline process

The multimodal fusion pipeline consists of three main components: (1) a custom Weighted ResNet for extracting features from dermatoscopic images that we define as DermiResNet, (2) a Clinical MLP for processing clinical metadata, and (3) a fusion module that combines the extracted features for final classification. The pipeline operates as follows:

- **Input**: Dermatoscopic images and clinical metadata are preprocessed and fed into the pipeline.
- **Feature extraction**: DermiResNet processes the images, while the Clinical MLP processes the metadata.
- **Fusion**: The extracted features are combined using one of several fusion techniques that are further discussed in the paper.
- **Classification**: The fused feature vector is passed through a Feed-forward Neural Network (FFN) to predict the skin lesion class.

**Figure 2:**
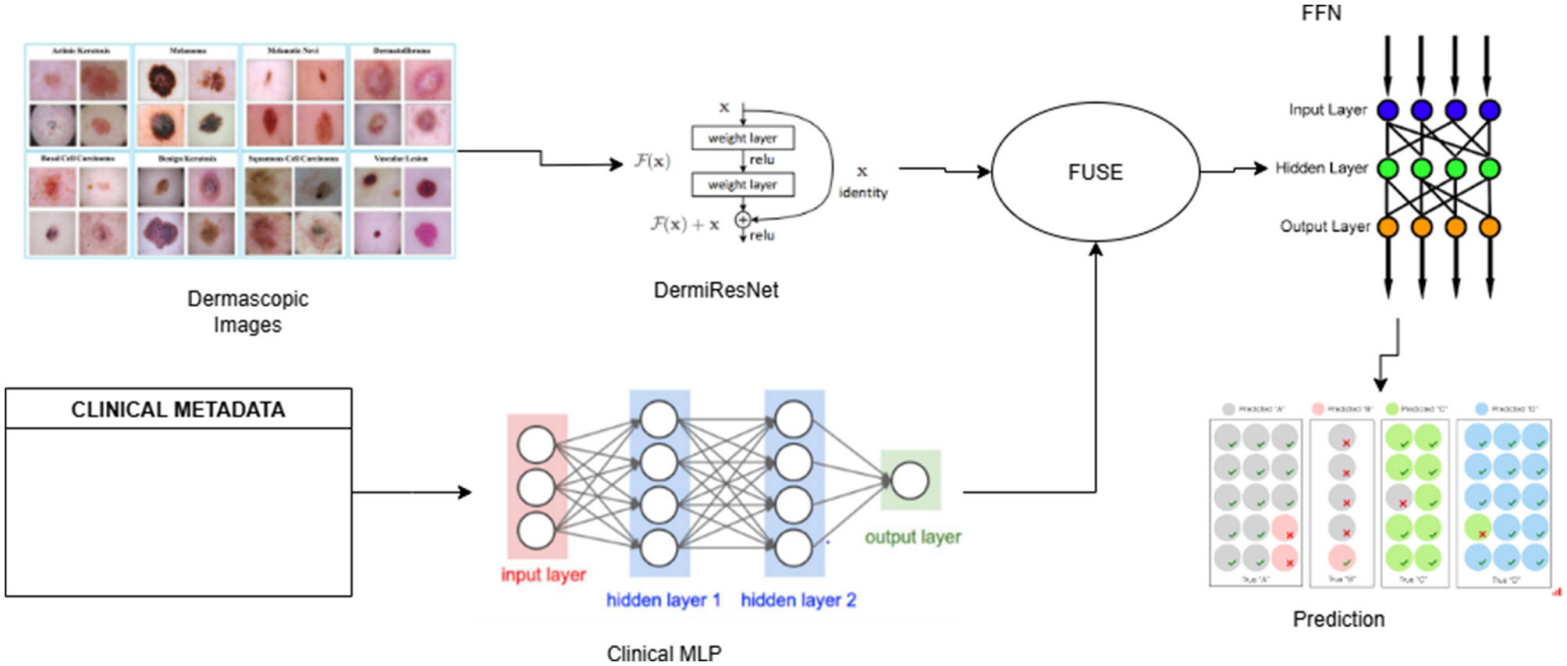
Overview of the multimodal fusion pipeline.

The details of all networks has been provided in the architecture section and the pipeline has been illustrated in Figure 2.

### 3.4 Architecture

#### 3.4.1 Clinical MLP

A **Multi-Layer Perceptron (MLP)** is a class of feedforward artificial neural networks composed of fully connected layers and nonlinear activation functions. MLPs are particularly well-suited for processing **structured tabular data** such as patient metadata, which lacks spatial structure and does not benefit from convolutional operations (Rumelhart et al., 1986).

In this study, we utilize a lightweight yet effective multi-layer perceptron (MLP) to process structured clinical metadata, including variables such as age, sex, and anatomical site of the lesion. The input is a 1D vector representation of all available metadata fields, which is first mapped to a 128-dimensional latent space via a fully connected layer with ReLU activation. This is followed by a second fully connected layer that expands the representation to 256 dimensions. This compact architecture is designed to extract meaningful latent embeddings from clinical features while being computationally efficient.

**Figure 3:**
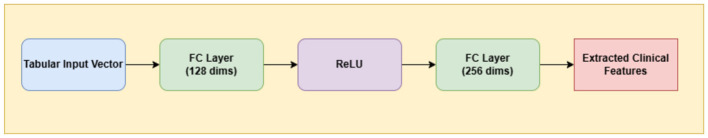
Architecture of the clinical MLP.

Figure 3 delineates the architectural pathway of the Clinical MLP, emphasizing its role in encoding patient metadata into latent space.

#### 3.4.2 DermiResNet

Residual Networks (ResNets), introduced to mitigate the degradation problem in deep architectures, extend conventional convolutional networks by learning residual mappings instead of direct functions (He et al., 2016). Rather than learning an unreferenced function, ResNets learn a residual mapping, which simplifies optimization and enables very deep architectures. Unlike AlexNet, ResNet is conceptually derived from the simpler and deeper VGG networks (Simonyan and Zisserman, 2015), but it introduces residual or skip connections that alleviate vanishing gradient issues during training.

A typical residual block includes two convolutional layers. If *x* is the input to a block, and *W*_1_, *W*_2_ are convolution kernels with non-linear activation σ, then the residual output is given by:


(1) $$\begin{array}{l} F\left(x\right)=W_{2}\cdot \sigma \left(W_{1}\cdot x\right) \end{array}$$

This results in the following expression for the block's output:


(2) $$\begin{array}{l} y=F\left(x\right)+x \end{array}$$

DermiResNet extends this formulation by introducing a learnable weight α for the skip connection:


(3) $$\begin{array}{l} y=F\left(x\right)+\alpha \cdot x \end{array}$$

Here, α ∈ ℝ is a learnable scalar parameter that determines the relative importance of the shortcut connection, enabling the model to adaptively weigh the residual and identity paths during training (Xu et al., 2024).

The architecture begins with a primary convolutional module (conv1), after which the network progresses through four sequential stages. Each stage consists of a downsampling convolutional unit and a corresponding residual unit. Across these stages, the number of feature channels is gradually increased (64 → 128 → 256 → 512), while spatial dimensions are reduced through stride-2 convolutions. Within each residual unit, two convolutional layers are used, each followed by batch normalization and LeakyReLU activation. To mitigate overfitting, dropout is applied in the later residual units (res3 and res4).

**Figure 4:**
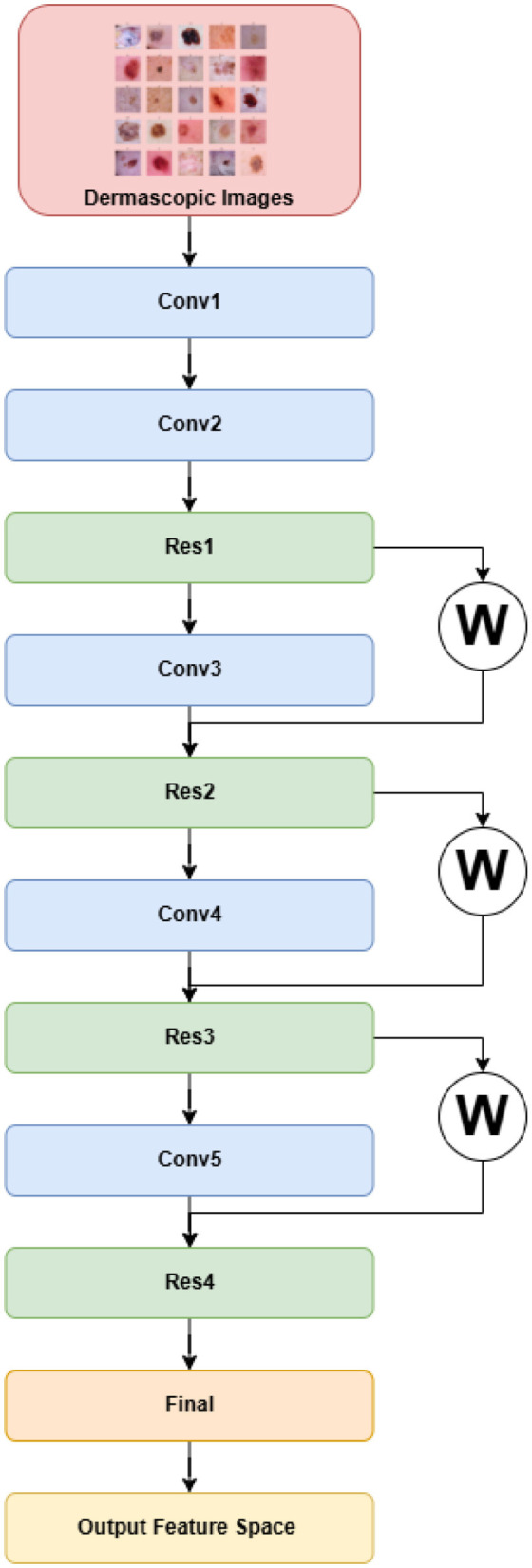
DermiResNet.

**Figure 5:**
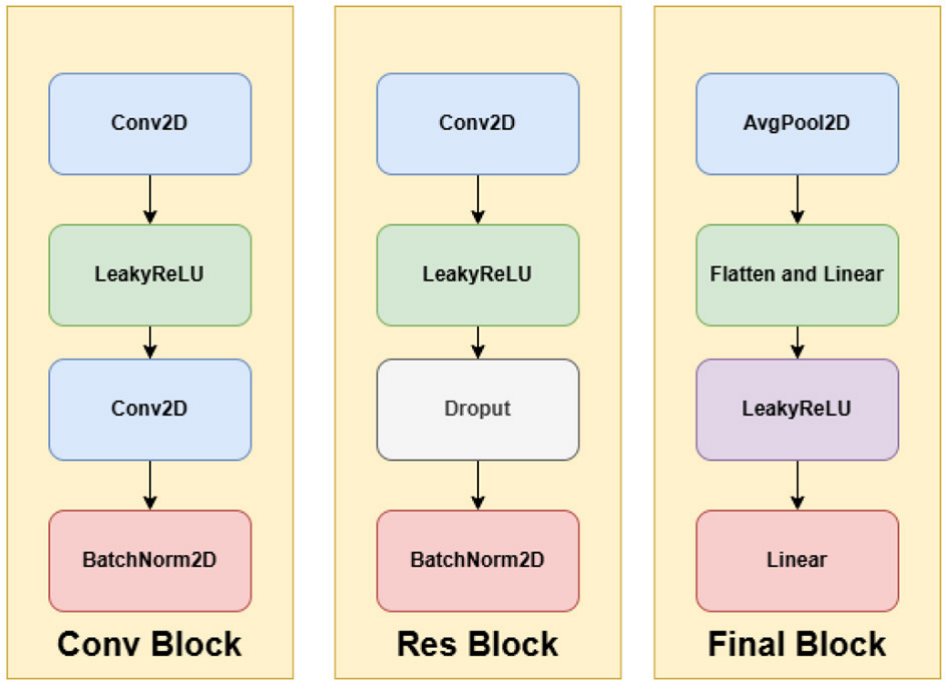
Blocks in DermiResNet.

The final classification head includes an adaptive average pooling layer, flattening, a two-layer fully connected network, and a softmax activation to output a 512-dimensional feature vector. The complete architecture is visualized in Figures 4, 5.

#### 3.4.3 Fusion block

In this layer, we fuse the extracted features from the DermiResNet (image features) and the Clinical MLP (clinical metadata features). The fusion process combines these multimodal features into a unified representation, which is then passed to a simple classifier for final prediction. The classifier consists of fully connected layers with ReLU activation functions, reducing the fused feature space to 7 output classes corresponding to the skin lesion types.

We explore several fusion techniques to combine the features effectively, including:

- Simple concatenation
- Weighted concatenation
- Hadamard product
- Tensor fusion
- Bilinear fusion
- Gated fusion
- Self-attention
- Cross-attention

Detailed descriptions of these fusion techniques, including their mathematical formulations and implementation, are provided in Section 3.5

### 3.5 Fusion details

Fusion involves integrating information from different sources or data modalities into a single, cohesive representation. This approach is especially valuable in **multimodal learning**, where inputs such as images, metadata, or text are combined to enhance model accuracy and robustness. In this study, we combine features produced by the **DermiResNet** (which yields 512-dimensional image embeddings) and the **Clinical MLP** (which produces 256-dimensional clinical metadata embeddings). The resulting fused representation serves as the input to the classification layer. The following subsections present and analyze several fusion strategies explored in our experiments, highlighting their relative strengths and performance.

#### 3.5.1 Simple concatenation

Simple concatenation involves merging features from different modalities by **stacking** them along a specific dimension. Given two feature vectors **x** ∈ ℝ^512^ (from the image model) and **y** ∈ ℝ^256^ (from the clinical model), the concatenated feature vector **z** ∈ ℝ^768^ is:


(4) $$\begin{array}{l} \text{z}=\left[\text{x};\text{y}\right] \end{array}$$

Equation 4 shows the simple concatenation of two feature vectors.

#### 3.5.2 Weighted concatenation

**Weighted concatenation** improves on simple concatenation by applying modality-specific weights. Instead of blindly stacking features, we scale each vector by a **learnable** scalar weight to reflect its importance (Ngiam et al., 2011; Kiela et al., 2018). Let *w*_1_, *w*_2_ ∈ ℝ be scalar weights applied to the 512D image vector **x** and 256D clinical vector **y**, respectively. The fused vector **z** ∈ ℝ^768^ is:


(5) $$\begin{array}{l} \text{z}=\left[w_{1}\cdot \text{x};w_{2}\cdot \text{y}\right] \end{array}$$

#### 3.5.3 Hadamard product fusion

The **Hadamard product**, or element-wise multiplication, combines two modalities by interacting their elements multiplicatively. Since this requires equal dimensions, we first project both **x** ∈ ℝ^512^ and **y** ∈ ℝ^256^ into a common latent space ℝ^256^. The fused representation **z** ∈ ℝ^256^ is given by:


(6) $$\begin{array}{l} \text{z}={\text{x}}^{\prime }⊙\text{y} \end{array}$$

Here, ${\text{x}}^{\prime }={\text{Linear}}_{512\to 256}\left(\text{x}\right)$, and ⊙ represents element-wise multiplication (Kim et al., 2017).

#### 3.5.4 Tensor fusion

**Tensor fusion** captures all possible interactions between modalities using an outer product, resulting in a second-order tensor (Zadeh et al., 2017). For **x** ∈ ℝ^512^ and **y** ∈ ℝ^256^, the fused tensor **Z** ∈ ℝ^512 × 256^ is:


(7) $$\begin{array}{l} \text{Z}=\text{x}⊗\text{y} \end{array}$$

This allows pairwise modeling of every feature from one modality with every feature from the other, at the cost of increased dimensionality.

#### 3.5.5 Bilinear fusion

**Bilinear fusion** is a feature interaction mechanism that combines information from two different modalities by explicitly modeling the **pairwise multiplicative interactions** between their respective features. Unlike simple concatenation or element-wise operations, bilinear fusion generates a richer and more expressive representation by learning how every feature from one modality interacts with every feature from the other. Conceptually, bilinear fusion captures second-order statistics between modalities—unlike first-order techniques such as concatenation, which only represent raw values. This is particularly valuable in multimodal tasks where the interplay between modalities is non-trivial and nonlinear (Fukui et al., 2016).

Given an image feature vector **x** ∈ ℝ^512^ and a clinical metadata feature vector **y** ∈ ℝ^256^, the bilinear fusion mechanism applies a set of bilinear mappings to produce a fused feature vector **z** ∈ ℝ^256^. Each dimension *z*_*i*_ of the output vector is computed using a learnable bilinear interaction:


(8) $$\begin{array}{l} z_{i}={\text{x}}^{⊤}{\text{W}}_{i}\text{y},\;\text{for }i=1,\ldots ,256 \end{array}$$

Here, each ${\text{W}}_{i}\in {\mathbb{R}}^{512\times 256}$ is a slice of the 3D learnable weight tensor $W\in {\mathbb{R}}^{256\times 512\times 256}$, such that:


(9) $$\begin{array}{l} \text{z}={\left[{\text{x}}^{⊤}{\text{W}}_{1}\text{y},\ldots ,{\text{x}}^{⊤}{\text{W}}_{256}\text{y}\right]}^{⊤} \end{array}$$

This results in a final fused representation **z** ∈ ℝ^256^, which is then passed through a fully connected layer for classification.

#### 3.5.6 Gated fusion

**Gated fusion** introduces a dynamic weighting mechanism that assigns different attention to modalities based on input features (Arevalo et al., 2020). The gating vector **g** ∈ ℝ^256^ is derived from a sigmoid function applied to a learnable affine combination of inputs:


(10) $$\begin{array}{l} \text{g}=\sigma \left({\text{W}}_{x}{\text{x}}^{\prime }+{\text{W}}_{y}\text{y}\right) \end{array}$$

The final fusion is:


(11) $$\begin{array}{l} \text{z}=\text{g}⊙{\text{x}}^{\prime }+\left(1-\text{g}\right)⊙\text{y} \end{array}$$

Here, ${\text{x}}^{\prime }={\text{Linear}}_{512\to 256}\left(\text{x}\right)$, and ⊙ denotes element-wise multiplication. This fusion strategy adaptively prioritizes modalities at an instance level.

#### 3.5.7 Self-attention fusion

**Self-attention** enables the model to attend to the most relevant parts of a single modality. Applied independently on each modality, it transforms the sequence of features **X** ∈ ℝ^*n*×*d*^ via attention weights:


(12) $$\begin{array}{l} \text{Z}=\text{softmax}\left(\frac{\text{Q}{\text{K}}^{⊤}}{\sqrt{d}}\right)\text{V} \end{array}$$

Where:


$$\text{Q}=\text{x}{\text{W}}_{Q},\;\text{K}=\text{x}{\text{W}}_{K},\;\text{V}=\text{x}{\text{W}}_{V}$$

Here, ${\text{W}}_{Q},{\text{W}}_{K},{\text{W}}_{V}\in {\mathbb{R}}^{d\times d}$ are learnable weight matrices. In this context, **Q** (Query) represents the features for which we want to find contextual relevance, **K** (Key) encodes the features to be compared against, and **V** (Value) holds the actual information to be aggregated. Self-attention enables each position in the feature sequence to attend to all positions, allowing the model to learn intra-modality dependencies. It is used before fusion to enhance modality-specific representations (Vaswani et al., 2017).

#### 3.5.8 Cross-attention fusion

**Cross-attention** aligns features between modalities by using one modality as **query** and the other as **key-value pairs**. For image features **X** ∈ ℝ^1 × 512^ and metadata features **Y** ∈ ℝ^1 × 256^, the query is derived from image features and the key/value from metadata:


(13) $$\begin{array}{l} \text{z}=\text{softmax}\left(\frac{{\text{Q}}_{x}{\text{K}}_{y}^{⊤}}{\sqrt{d}}\right){\text{V}}_{y} \end{array}$$

Where:


$${\text{Q}}_{x}=\text{x}{\text{W}}_{Q},\;{\text{K}}_{y}=\text{y}{\text{W}}_{K},\;{\text{V}}_{y}=\text{y}{\text{W}}_{V}$$

In cross-attention, **Q**_*x*_ represents the query derived from the primary modality (e.g., image features), which is seeking relevant complementary information. **K**_*y*_ and **V**_*y*_ are the key and value vectors derived from the secondary modality (e.g., clinical metadata), where the key determines alignment and the value contributes the corresponding context. This fusion allows modality **X** to selectively attend to modality **Y**, creating cross-modal representations that are aligned and context-aware (Lu et al., 2019).

### 3.6 Experimental set-up

#### 3.6.1 Hardware and software configuration

The hardware and software specifications used for the experiments are summarized in Table 4.

**Table 4:** Hardware and software configuration.

**Component**	**Specification**
GPU	NVIDIA GeForce RTX 4060
Processor	AMD Ryzen 7 7800X3D
Memory	16GB DDR4 RAM
Operating System	Linux Mint 21.1
CUDA	Enabled

#### 3.6.2 Training configuration

The specific training configuration, which outlines hyperparameters and other details, is documented in Table 5. Each model was trained for an estimated duration of 2 h.

**Table 5:** Training configuration.

**Parameter**	**Value**
Batch size	64
Number of epochs	100
Learning rate	0.001
Optimizer	Adam
Loss function	Cross-entropy loss

### 3.7 Evaluation metrics

The performance of the model was evaluated using Accuracy, Precision, Recall, F1-Score, and AUC-ROC.

## 4 Result analysis and discussion

### 4.1 Ablation studies

To assess the contribution of each modality to the overall model performance, we first evaluated the individual models trained separately on clinical metadata and dermatoscopic images. The results of these experiments are summarized in Table 6.

**Table 6:** Performance of individual models in ablation studies.

**Model**	**Accuracy**	**Precision**	**Recall**	**F1-Score**
Clinical MLP (metadata only)	77.0%	0.76	0.75	0.76
DermiResNet (image only)	92.0%	0.91	0.92	0.91

The Clinical MLP achieved an accuracy of **77.0%**, indicating that clinical metadata alone offers moderate predictive capability. However, the DermiResNet, trained exclusively on dermatoscopic images, achieved a significantly higher accuracy of **92.0%**, showcasing the superior discriminative power of visual data for skin lesion classification. This result aligns with the diagnostic process commonly employed by dermatologists, where visual inspection of skin lesions is typically prioritized over metadata analysis for accurate classification (Dinnes et al., 2018).

### 4.2 Multimodal fusion performance

We evaluated the performance of various fusion techniques, including simple concatenation, weighted concatenation, Hadamard product, tensor fusion, bilinear fusion, gated fusion, self-attention, and cross-attention. The results are summarized in Table 7.

**Table 7:** Performance of multimodal fusion techniques.

**Fusion technique**	**Accuracy**	**Precision**	**Sensitivity (recall)**	**F1-score**	**Specificity**
Simple concatenation	96.5%	0.93	0.93	0.93	0.97
Weighted concatenation	97.15%	0.97	0.97	0.97	0.98
Hadamard product	98.85%	0.97	0.97	0.97	0.99
Tensor fusion	96.52%	0.96	0.96	0.96	0.97
Bilinear fusion	98.76%	0.98	0.98	0.98	0.99
Gated fusion	93.0%	0.93	0.93	0.92	0.91
Self-attention	92.70%	0.92	0.93	0.92	0.90
Cross-attention	98.86%	0.98	0.98	0.98	0.99

The comparison of different **multimodal fusion methods** provides profound insights into the design of AI-powered medical diagnostic systems. From among the methods, two high-performance methods are notable: **Cross-attention** and the **Hadamard product**, both of which deliver near state-of-the-art performance with respective accuracies of **98.86%** and **98.85%**.

**Cross-attention**, one of the high-performance and intricate methods, performs exceptionally well by dynamically weighting and matching modalities' features. Through the computation of attention scores between dermatoscopic image features and clinical metadata, it dynamically concentrates on the most discriminative data for every input. This imitates the subtle diagnosis reasoning of skilled clinicians to a great extent. For example, when visual features are uncertain (e.g., look-alike lesions), Cross-Attention uses contextual information like patient age or lesion site to sharpen its prediction. This capacity to represent high-grained, adaptive interactions makes it particularly useful for sophisticated diagnostic tasks such as skin lesion classification (Ou et al., 2022).

**Hadamard product**, another high-performance method but relatively less complex in design, combines features through element-wise multiplication. It extracts localized interactions across modalities, for example, texture-lesion vs. age correlations, with remarkable performance. Nevertheless, in contrast to Cross-Attention, it does not have the dynamic feature importance adaptation capability that may limit its effectiveness in extremely ambiguous or nonlinear situations.

Conversely, certain **sophisticated techniques performed poorly**. **Gated fusion** (**93.0% accuracy**) and **Self-attention fusion** (**92.70% accuracy**) performed poorly despite their complex architectural design. Gated Fusion adds more learnable parameters in the form of gating mechanisms, which can lead to heightened overfitting risks and tougher training, particularly with small data sizes. Self-Attention is incredibly strong in a single modality but might miss key inter-modality dependencies when utilized stand-alone, thus reducing its multimodal performance.

Surprisingly, also simple approaches can perform well. **Weighted concatenation**, a low-complexity method, performed **97.15% accuracy**. By using static or learnable weights for every modality prior to concatenation, it is good at balancing interpretability, stability, and performance. Without modeling high-level feature interactions, its simplicity and stability make it a very practical solution for most clinical scenarios.

These findings highlight an important point: **algorithmic complexity is no guarantee of better performance**. The selection of fusion strategy must be informed by the particular properties of the dataset and clinical application, striking a balance between accuracy, interpretability, and computational cost.

In conclusion, while sophisticated fusion mechanisms such as **Cross-attention** deliver state-of-the-art performance by dynamically aligning visual and clinical modalities, our results demonstrate that in some scenarios, simpler techniques like Weighted Concatenation are highly competitive offering comparable accuracy with significantly lower computational overhead. This makes them well-suited for practical deployment in real-world medical settings, particularly where resources are constrained or rapid inference is essential. Cross-attention, on the other hand, proves most valuable in cases involving complex diagnostic patterns—such as ambiguous lesions or subtle correlations between metadata and visual features, where adaptive modality interaction becomes critical.

Ultimately, the success of a multimodal fusion approach lies not merely in algorithmic sophistication but in its ability to replicate the holistic, context-aware diagnostic reasoning employed by clinicians. The choice of fusion strategy should thus be guided by the specific clinical setting, data complexity, and resource constraints, striking a balance between accuracy, interpretability, and efficiency.

### 4.3 Confusion matrix analysis

**Figure 6:**
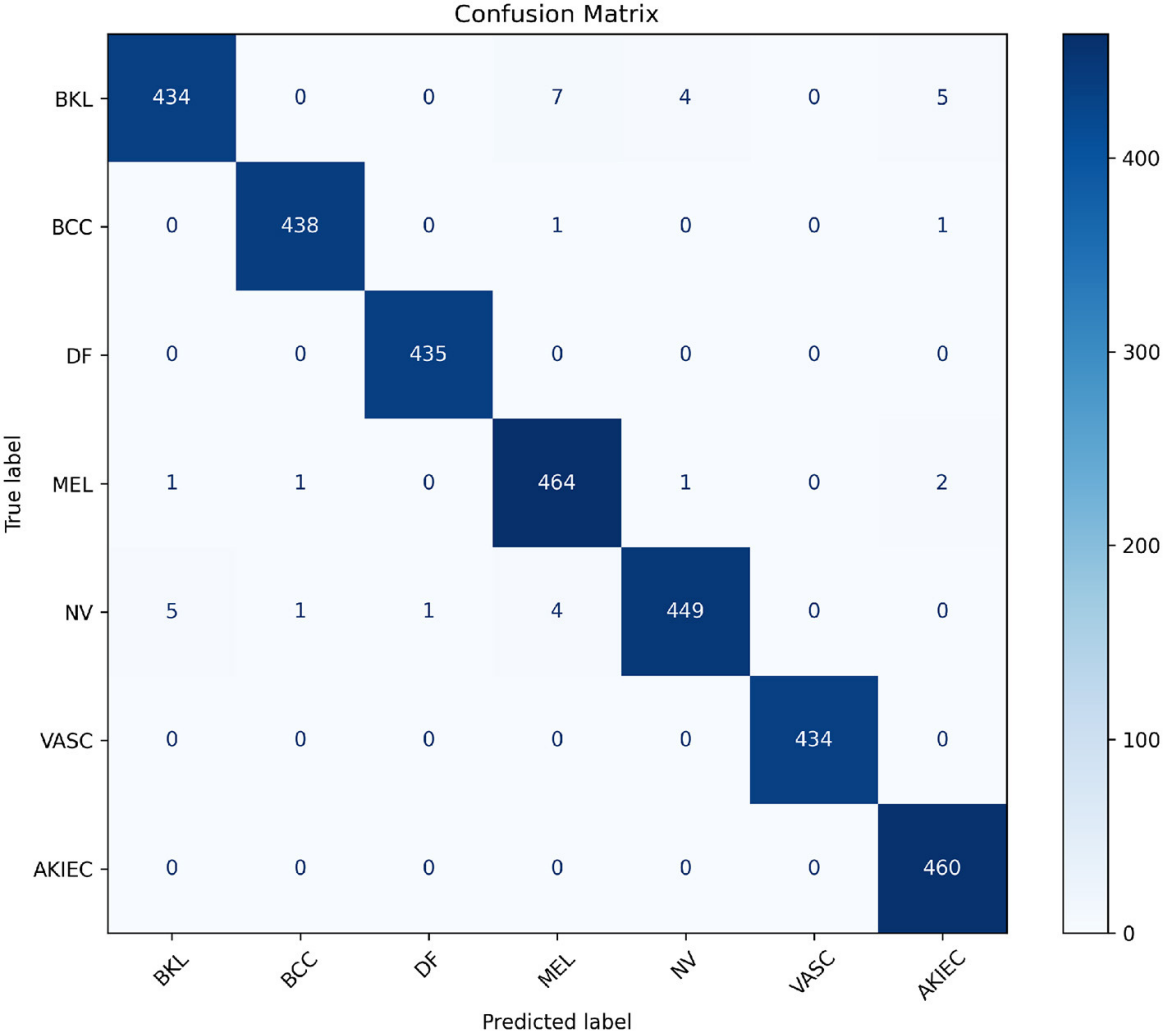
Visualization of the confusion matrix for the best-performing Cross-attention model. This heatmap helps identify specific misclassifications and overall model behavior across classes.

To further analyze the performance of the best-performing fusion model (Cross-attention), we present its confusion matrix in Figure 6. The matrix shows the number of correct and incorrect predictions for each class.

The confusion matrix highlights the performance of the model across various skin cancer classes. Notably, the diagonal entries, which represent correctly classified instances, dominate, demonstrating strong overall performance.

The model achieves near-perfect classification for classes like **DF**, **VASC**, and **AKIEC**, with minimal or no misclassifications. This suggests that these classes are well-represented in the training data and exhibit distinct features, allowing the model to identify them with high confidence.

However, there are minor misclassifications, particularly between similar-looking lesion types such as **BKL** and **MEL** or **NV**. For instance, 7 cases of **BKL** are misclassified as **MEL**, and 5 as **AKIEC**, indicating some overlap in visual or clinical features. Similarly, 4 cases of **NV** are mistaken for **MEL**, which is expected given their subtle differences and shared features in certain instances.

The small number of misclassifications in **BCC** and **MEL** classes suggests the model handles malignant lesions well but still requires improvements to reduce errors in high-stakes scenarios.

Overall, the model demonstrates high classification accuracy with room for improvement in distinguishing between lesion types with overlapping visual or clinical characteristics.

### 4.4 ROC-AUC curve

**Figure 7:**
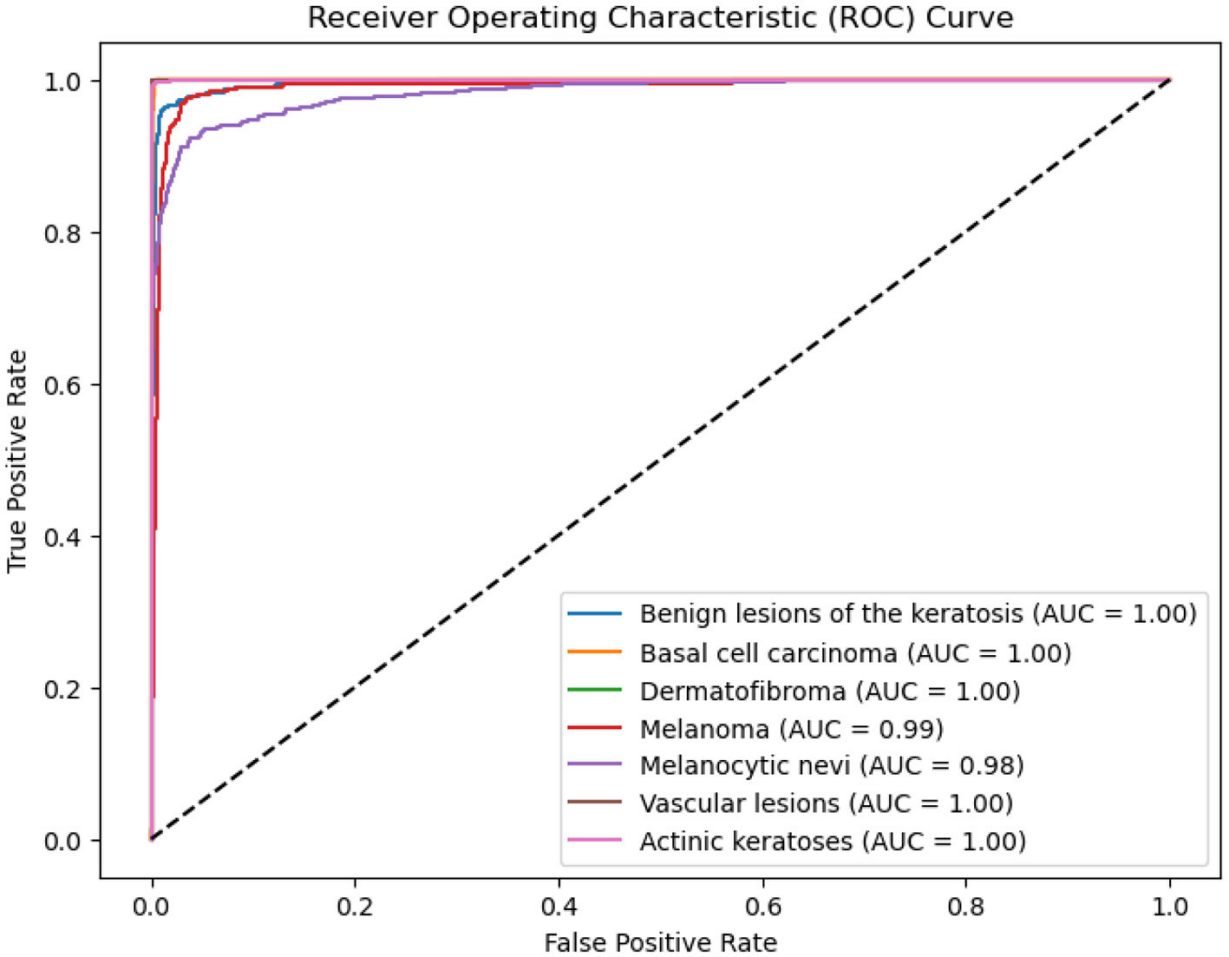
ROC-AUC curve for melanoma, nevi, and other classes.

The ROC-AUC scores for different classes are presented in the figure below. The model achieved a score of 0.99 for MEL, 0.98 for NV, and 1.0 for the remaining classes, as shown in the ROC curve (Figure 7).

These high AUC values suggest that the model is capable of effectively distinguishing between the classes. The results indicate that there is no sign of underfitting or overfitting, with the model generalizing well and avoiding excessive fitting to noise in the training data.

### 4.5 Explainability

Explainability is a foundation of applying machine learning models in healthcare. Although deep learning models tend to exhibit state-of-the-art performance, their “black-box” nature is highly problematic in the healthcare domain. Physicians and medical experts need interpretable models for them to comprehend the rationale of predictions, making diagnoses not only correct but also clinically reasonable. Lack of transparency can translate to mistrust, preventing the implementation of AI systems into actual healthcare workflows.

Black-box models that give no clue about their reasoning are especially troubling in high-risk areas such as dermatology. For example, a model can be highly accurate by leveraging spurious correlations or data artifacts instead of clinically significant features, leading to serious failures when deployed in more diverse or unseen clinical environments (Zech et al., 2018). Explainability closes this gap by illuminating how a model comes to a decision, allowing doctors to verify its reasoning and spot potential errors or biases (Amann et al., 2020).

This research emphasize explainability so that the multimodal fusion model is not just precise but also reliable and comprehensible. Through the use of visual explanations (Grad-CAM) coupled with relevance analysis of clinical features, an integrated understanding of the decision-making process of the model is presented.

#### 4.5.1 Grad-CAM

Grad-CAM is a powerful technique for visualizing the regions of an image that are most influential in a model's decision. It extends the Class Activation Mapping (CAM) approach by using gradient information to weight the importance of feature maps, making it applicable to a wider range of architectures, including those without global average pooling layers.

**Mathematical formulation**: Let *A*^*k*^ be the activation map of the *k*-th channel in the target convolutional layer, and let *y*^*c*^ be the score for class *c*. The weight $\alpha_{k}^{c}$ for the *k*-th channel is computed as the global average of the gradients of *y*^*c*^ with respect to *A*^*k*^:


$$\alpha_{k}^{c}=\frac{1}{Z}\underset{i}{\sum }\underset{j}{\sum }\frac{\partial y^{c}}{\partial A_{ij}^{k}},$$

where *Z* is the number of pixels in the activation map. These weights capture the importance of each feature map for the target class.

The Grad-CAM heatmap *L*^*c*^ is then obtained by a weighted combination of the activation maps, followed by a ReLU function:


$$L^{c}=\text{ReLU}\left(\underset{k}{\sum }\alpha_{k}^{c}A^{k}\right).$$

The ReLU function ensures that only positive influences are considered, as negative values are not relevant for the target class (Selvaraju et al., 2017).

Grad-CAM indicates areas of the image that the model considers most significant for its prediction. For instance, in images obtained with dermatoscopy, these areas may be lesion boundaries, texture, or color transitions. By projecting these areas, Grad-CAM opens a window to the model's “thinking process,” allowing physicians to ensure that the AI system is concentrating on clinically relevant features (Jaworek-Korjakowska et al., 2021).

#### 4.5.2 Analysis of grad-CAM results for cross-attention

To understand the decision-making process of the top-performing model, which utilizes a cross-attention fusion approach, Grad-CAM visualizations were employed. This technique facilitates the interpretation of the model's predictions by identifying key areas in dermatoscopic images that most strongly influence the results. Such *post-hoc* explainability is especially valuable in medical contexts, as it allows clinicians to confirm that the model's predictions are grounded in clinically significant features.

The cross-attention fusion mechanism enhances the interpretability of the model by dynamically aligning and integrating features from different modalities or scales. Unlike simpler fusion techniques, cross-attention computes attention weights between modalities, enabling the model to emphasize the most diagnostically relevant interactions. While Grad-CAM visualizations help interpret the image-based decision process by highlighting regions of importance in dermatoscopic inputs, they are limited to visual modalities. To gain a holistic understanding of the model's reasoning, particularly for clinical metadata, we later discuss Clinical Feature Relevance Scores, which complement Grad-CAM by providing insight into how non-image features influence predictions. This combined interpretability offers a more comprehensive explanation of the model's diagnostic behavior.

**Figure 8:**
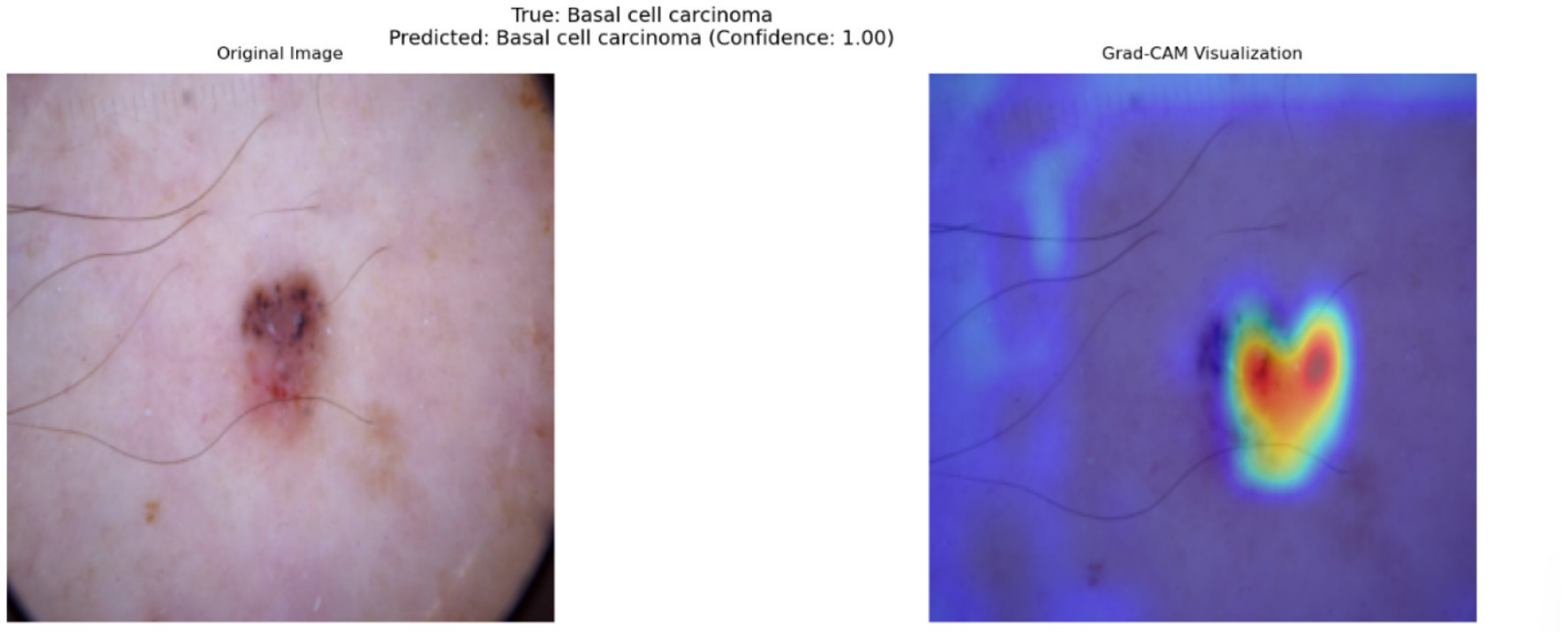
Grad-CAM visualization for a dermatoscopic image classified as Basal Cell Carcinoma. The left panel shows the original image, while the right panel highlights clinically significant regions contributing to the model's prediction. Red regions indicate high importance, and blue regions indicate low importance.

Figure 8 provides an illustrative example of a Grad-CAM heatmap overlaid on an input dermatoscopic image. The model predicts the lesion as BCC with a confidence score of 1, focusing primarily on the lesion's irregular borders and regions of heterogeneous pigmentation. These features are clinically significant as BCC are characterized by asymmetry, border irregularity, and color variation (Puckett et al., 2025).

In this specific example, the heatmap demonstrates that the model successfully identifies features associated with malignancy while ignoring irrelevant artifacts, such as hair strands and uniform skin areas. The model's ability to focus on clinically relevant features is a direct outcome of the cross-attention fusion mechanism, which dynamically aligns and weights features from different modalities. By doing so, the model achieves a higher level of alignment with dermatological practices, where lesion borders and internal variations are critical for diagnosis.

As discussed earlier, the model occasionally misclassifies BKL as MEL due to overlapping visual characteristics. One contributing factor is that both lesion types can exhibit irregular pigmentation, asymmetric structures, and varying border definitions, which are also key diagnostic markers for MEL. This confusion is particularly evident in cases where keratosis presents with darker pigmentation and irregular borders, mimicking features of malignant lesions.

**Figure 9:**
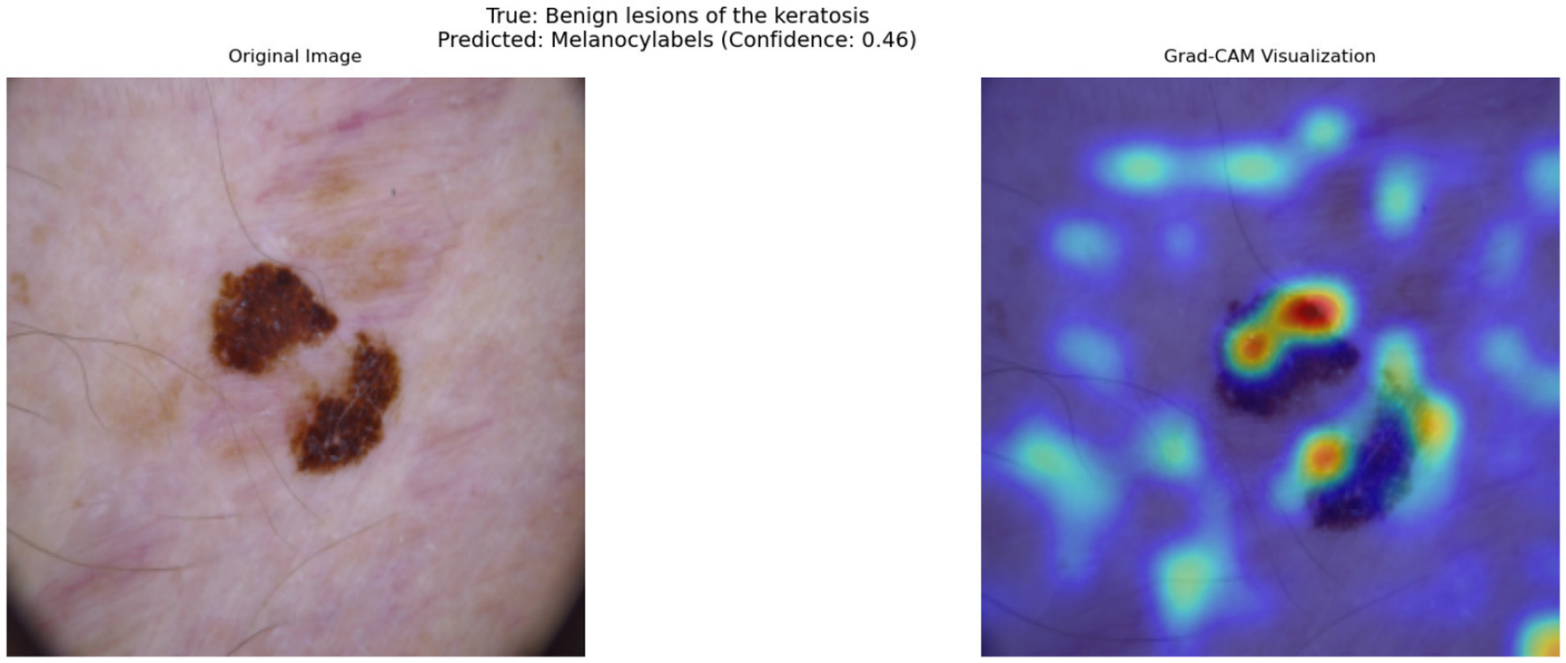
Grad-CAM visualization of a misclassified Benign Keratosis case. The model incorrectly predicts this lesion as Melanocytic (MEL) with a confidence of 0.46.

Figure 9 illustrates such a misclassification, where a BKL lesion has been incorrectly predicted as MEL with a confidence score of 0.46. The left panel shows the original image, while the right panel presents the corresponding Grad-CAM heatmap, highlighting the regions that influenced the model's decision.

From the heatmap, it is evident that the model assigns high importance (red/yellow regions) to darker pigmented areas and irregular structures within the lesion. This suggests that the model's decision boundary between benign and malignant lesions is influenced primarily by pigmentation and border irregularity, which are not always exclusive to melanoma.

Beyond this example, Grad-CAM visualizations across a wide range of test cases reveal consistent patterns in the model's behavior. The model often prioritizes irregular lesion borders and regions of color variation, which are crucial for distinguishing malignant lesions from benign ones. In cases of BCC, the heatmaps highlight central regions with ulceration or shiny surfaces, further reinforcing the model's alignment with clinical indicators. This interpretability is invaluable for real-world deployment, as it allows clinicians to confirm that the model's focus areas correspond to meaningful diagnostic features.

The insights provided by Grad-CAM are directly correlated with the model's strong quantitative performance. For example, the regions identified by the heatmaps frequently align with features responsible for the model's high sensitivity and specificity, particularly in challenging cases of melanoma and basal cell carcinoma. This combination of performance metrics and visual interpretability underscores the potential of the cross-attention fusion-based model as a trustworthy diagnostic aid.

**Figure 10:**
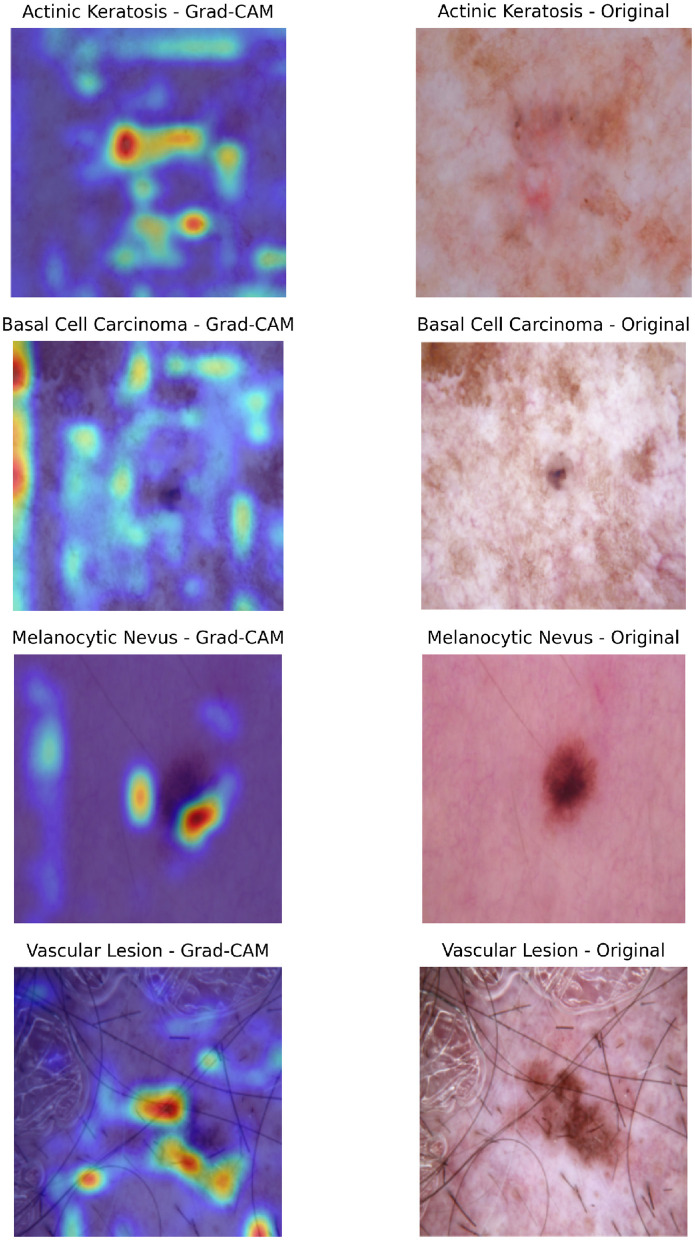
Composite visualization of Grad-CAM outputs for selected classification cases. For each sample, the left panel shows the Grad-CAM heatmap overlaid on the dermatoscopic image, highlighting regions the model attended to during prediction. The right panel displays the original dermatoscopic image with the ground truth label indicated. This layout enables intuitive interpretation of model focus and correctness of attention alignment.

Additional Grad-CAM visualizations for Cross-attention are provided in Figure 10, showcasing further examples of model attention across different lesion types and cases.

#### 4.5.3 Clinical feature relevance

To assess the instance-specific relevance of clinical features, we employed an attribution-based approach using Integrated Gradients (IG) (Sundararajan et al., 2017). IG is a path-based attribution method that quantifies feature importance by computing the integral of gradients along an interpolation path from a baseline input to the actual input.

Given an input clinical feature vector *x* ∈ ℝ^*d*^ and a baseline vector *x*′ ∈ ℝ^*d*^, the integrated gradient for the *i*^*th*^ feature is computed as:


(14) $$\begin{array}{l} IG_{i}\left(x\right)=\left(x_{i}-x_{i}^{\prime }\right)\times \int_{\alpha =0}^{1}\frac{\partial f\left(x^{\prime }+\alpha \left(x-x^{\prime }\right)\right)}{\partial x_{i}}d\alpha , \end{array}$$

where *f*(·) represents the model's prediction score for the target class. The integral is approximated using a summation over discrete steps:


(15) $$\begin{array}{l} IG_{i}\left(x\right)\approx \left(x_{i}-x_{i}^{\prime }\right)\times \frac{1}{S}\overset{S}{\underset{s=1}{\sum }}\frac{\partial f\left(x^{\prime }+\frac{s}{S}\left(x-x^{\prime }\right)\right)}{\partial x_{i}}, \end{array}$$

where *S* is the number of interpolation steps.

For each instance, we set the baseline *x*′ as a zero vector, representing the absence of clinical features. The integrated gradients were computed over *S* = 50 steps, and the absolute values of the attributions were taken as feature importance scores. These scores were then normalized to the range [0, 1] to facilitate interpretability.

The resultant feature relevance scores highlight the contribution of individual clinical attributes to the model's prediction. Features with higher attributions indicate stronger influence on the classification decision, thereby providing insights into the model's reliance on clinical metadata.

#### 4.5.4 Clinical feature relevance analysis

For a specific instance of **NV**, we analyzed the relative importance of clinical features using our best-performing Cross-Attention fusion model. As shown in Table 8, the top features with high importance include **localization** (scalp, foot, neck), and **diagnostic methods** (consensus, follow-up, confocal). These features likely play a significant role due to the distinct characteristics of lesions in these locations and the reliability of the diagnostic methods. Features with moderate importance, such as localization (face, genital) and diagnosis type (histopathology), contribute to predictions but are less critical. Interestingly, **age** and certain locations (e.g., ear, lower extremity) show low importance, suggesting they have minimal influence on the model's predictions for this instance. The balanced impact of **sex** (both male and female) indicates that while it influences outcomes, it is not among the strongest predictors. Overall, localization emerges as the most relevant feature, aligning with real-world clinical practice where lesion location is a key diagnostic factor.

**Table 8:** Instance-specific clinical feature importance for a melanocytic nevus instance.

**Clinical feature**	**Relative importance**
Dx type: follow-up	1.00
Localization: hand	0.80
Localization: scalp	0.75
Localization: neck	0.70
Localization: acral	0.65
Localization: lower extremity	0.60
Localization: chest	0.55
Localization: unknown	0.50
Localization: abdomen	0.45
Localization: genital	0.40
Sex: male	0.35
Dx type: consensus	0.30
Sex: unknown	0.25
Localization: upper extremity	0.20
Dx type: confocal	0.15
Localization: trunk	0.12
Localization: face	0.10
Sex: female	0.08
Localization: back	0.06
Localization: ear	0.04
Localization: foot	0.03
Age	0.02
Dx type: histopathology	0.01

Across various lesion types, we observed that **localization** consistently emerged as the most important clinical feature, underscoring its critical role in skin lesion diagnosis. This aligns with real-world clinical practice, where the anatomical location of a lesion is a key diagnostic factor due to its correlation with sun exposure, skin type, and lesion characteristics.

In cases of **MEL**, **localization** (e.g., back, face) was the top predictor, reflecting the higher prevalence of malignant lesions in sun-exposed areas. Additionally, **age** and **gender** showed moderate influence, consistent with their established roles as risk factors for melanoma. Older patients and males exhibited a higher likelihood of malignancy, further validating the model's alignment with epidemiological trends (Raimondi et al., 2020; Garbe et al., 2021).

For **BCC**, the model again prioritized **localization** (e.g., face, neck), as these areas are most susceptible to UV damage (Marzuka and Book, 2015). Diagnostic methods such as **histopathology** also played a significant role, as BCC is often confirmed through biopsy. Interestingly, **age** showed a stronger influence for BCC compared to other lesion types, likely due to the cumulative effect of UV exposure over time (Seretis et al., 2025).

In cases of **AKIEC**, **localization** (e.g., face, scalp) remained the most important feature, as AKIEC lesions are strongly associated with chronic sun exposure (Reinehr and Bakos, 2019). The model also highlighted the importance of **diagnostic methods** (e.g., follow-up, confocal microscopy), reflecting the need for repeated evaluations to monitor these precancerous lesions.

For **DF**, a benign lesion, **localization** (e.g., lower extremities) was again the dominant feature, while **age** and **gender** had minimal influence. This suggests that the visual appearance and location of DF lesions are more critical for diagnosis than demographic factors (Han et al., 2011).

Finally, in cases of **VASC**, **localization** (e.g., face, trunk) was the most influential feature, as these lesions often appear in specific anatomical regions (Mulligan et al., 2014). The model also relied heavily on **dermoscopic examination**, highlighting the importance of visual data for diagnosing vascular lesions.

## 5 Conclusion

The effectiveness of multimodal fusion methods in improving skin lesion classification performance is well illustrated through this study. By combining clinical metadata with dermatoscopic images, the model reached state-of-the-art accuracy, with the **Cross-attention** and **Hadamard product** methods reaching almost **99% accuracy**. Importantly, these accuracies were reached with a basic laptop setup, without the necessity for specialized Neurap Processing Units (NPUs) or workstations, making the proposed method efficient and accessible. This is especially important for resource-limited settings, where sophisticated computational facilities might not be easily accessible.

The strength of these fusion methods rests in their capacity to merge complementary information from visual and clinical data, emulating the comprehensive diagnostic reasoning that seasoned clinicians often exhibit. The **Cross-attention** method, specifically, performed exceptionally well by dynamically correlating and weighting features from both modalities to allow the model to concentrate on the most discriminative information per input. Likewise, the **Hadamard product** also performed well by appropriating local modality relationships through element-wise multiplication of feature vectors.

These results highlight the need for carefully choosing fusion techniques depending on the specific characteristics of the dataset and clinical context. Though more sophisticated methods like Cross-Attention present better performance in challenging scenarios, simpler approaches such as **Weighted concatenation** offer a useful trade-off between accuracy and computational expense.

### 5.1 Limitations and future directions

Although the findings of this study highlight the capability of multimodal fusion to improve skin lesion classification, it is essential to recognize various limitations requiring future work and improvement.

- **Computational resource requirements**: While the adopted methodologies illustrate feasibility on common computing hardware, the inherent computational intensity of sophisticated fusion methods, specifically tensor fusion and cross-attention, poses a significant computational burden. Future studies should focus on developing and utilizing optimization techniques. Model pruning, quantization, and knowledge distillation are some techniques that need to be explored to reduce computational overhead without compromising diagnostic performance.
- **Generalizability across diverse populations**: The use of the HAM10000 dataset, as comprehensive as it is, may not perfectly capture the heterogeneity of skin lesions observed in real-world clinical practice. Therefore, the generalizability of the model to diverse patient populations remains an important challenge. Future research should include external validation by evaluating performance on datasets such as ISIC and Pedro Hispano-2 (PH2). Additionally, expanding training data with a broader range of clinical and demographic variables is crucial to enhancing the model's robustness and validity.
- **Integration of extensive clinical data**: The predictive capability of the present model is constrained by the limited range of clinical features available in the HAM10000 dataset. To address this, future research should focus on integrating more comprehensive clinical data. This includes patient medical histories, genetic predisposition, and laboratory test results, which collectively contribute to a more holistic diagnostic analysis.
- **Improving model interpretability for clinical trust**: Clinical adoption of AI-based diagnostic tools is contingent on their interpretability. Although Grad-CAM provides a visual interpretation of feature importance, a deeper understanding of the model's decision-making process is necessary. Future studies should explore advanced Explainable AI (XAI) techniques, such as combining Grad-CAM with clinical feature relevance analysis or developing hybrid models that provide both visual and textual explanations.
- **Refinement of fusion methodologies**: The success of multimodal fusion depends on the selection and optimization of appropriate techniques. Future research should explore adaptive fusion methods that dynamically adjust based on input features. Additionally, investigating ensemble fusion techniques that leverage the strengths of multiple fusion strategies could lead to significant improvements in diagnostic accuracy.
- **Validation in real-world clinical environments**: To assess the practical effectiveness of the proposed system, rigorous validation in real-world clinical settings is essential. Future studies should emphasize real-time deployment of the model in diagnostic workflows, ensuring close collaboration with dermatologists and healthcare professionals to address implementation challenges and optimize the system based on real-world feedback.
- **Handling class imbalance and rare phenotypes**: The misclassification of rare transitions, such as melanoma being classified as nevus or benign keratosis, underscores the need for better handling of class imbalances and subtle feature variations. Beyond basic augmentation, future research should investigate more advanced strategies, such as:

• *Focal Loss*, which down-weights easy examples and focuses training on hard negatives, improving detection of minority classes (Lin et al., 2020).
• *Synthetic oversampling using GANs*, such as Deep Convolutional Architectures for Image Synthesis (DCGAN) or Style-Based Image Generation Networks (StyleGAN2), to generate realistic lesion images for underrepresented classes like dermatofibroma or vascular lesions (Frid-Adar et al., 2018; Mutepfe et al., 2021).

- **Improving preprocessing resilience**: The tendency of the model to focus on non-essential regions, such as hair strands or glossy skin, highlights the need for more robust preprocessing techniques. Enhancing hair removal algorithms and contrast normalization strategies is crucial to eliminating distractions and improving the model's reliability in assessing key lesion features.
- **Color constancy and harmonization**: Variations in lighting and acquisition devices can lead to inconsistent image appearance. Future work should explore *color constancy algorithms* to normalize illumination conditions across samples. Techniques like *Shades of Gray, Gray World*, and *Learning-Based Color Constancy* could significantly reduce lighting-induced variance (Bianco and Cusano, 2019; Barnard et al., 2002). This harmonization is critical for enhancing cross-device robustness in clinical deployment.
- **Cross-referencing segmentation with Grad-CAM**: While Grad-CAM provides valuable insights into model attention, it does not guarantee alignment with lesion boundaries. Future work should involve cross-referencing Grad-CAM heatmaps with lesion segmentation masks [e.g., generated using the **Segment Anything Model (SAM)** (Kirillov et al., 2023)] to verify that the model is focusing on diagnostically relevant regions. This integration could improve both model explainability and diagnostic reliability.

This research lays a strong foundation for applying multimodal fusion in skin lesion classification. By addressing the identified limitations and exploring the proposed future directions, we can advance the development of precise, efficient, and clinically viable AI-driven diagnostic systems, ultimately leading to improved outcomes for patients with dermatological conditions.

## Data Availability

Publicly available datasets were analyzed in this study. This data can be found here: https://www.kaggle.com/datasets/kmader/skin-cancer-mnist-ham10000 (Scott Mader, 2018).
